# Randomized Trial on the Effects of a Group EMDR Intervention on Narrative Complexity and Specificity of Autobiographical Memories: A Path Analytic and Supervised Machine-Learning Study

**DOI:** 10.3390/ijerph19137684

**Published:** 2022-06-23

**Authors:** Andrea Poli, Angelo Gemignani, Mario Miccoli

**Affiliations:** 1 Department of Clinical and Experimental Medicine, University of Pisa, 56126 Pisa, Italy; mario.miccoli2020@virgilio.it; 2 Department of Surgical, Medical and Molecular Pathology and of Critical Care Medicine, University of Pisa, 56126 Pisa, Italy; angelo.gemignani@unipi.it

**Keywords:** EMDR, childhood, psychological trauma, autobiographical memory, distress, cognition, narrative complexity, narrative specificity

## Abstract

Narratives of autobiographical memories may be impaired by adverse childhood experiences, generating narrative fragmentation and increased levels of perceived distress. Eye movement desensitization and reprocessing (EMDR) proved to be an effective treatment to overcome traumatic experiences and to promote coherent autobiographical narratives. However, the specific mechanisms by which EMDR promotes narrative coherence remains largely unknown. We conducted a randomized controlled pilot trial (ClinicalTrials.gov Identifier NCT05319002) in a non-clinical sample of 27 children recruited in a primary school. Participants were randomly assigned to the experimental and control groups. The experimental group underwent a three-week group EMDR intervention. Subjective unit of distress (SUD), validity of cognition (VoC), classification of autobiographical memories, narrative complexity and specificity were assessed before and after the group EMDR intervention. The group EMDR intervention was able to improve SUD and VoC scales, narrative complexity and specificity, and promoted the classification of autobiographical memories as relational. The path analysis showed that SUD was able to predict VoC and narrative specificity, which, in turn, was able to predict both narrative complexity and the classification of autobiographical memories as relational. Machine-learning analysis showed that random tree classifier outperformed all other models by achieving a 93.33% accuracy. Clinical implications are discussed.

## 1. Introduction

There is plenty of research, generated over more than two decades, which shows a link between traumatic event exposure and the specificity of autobiographical memories [1], fragmented trauma memories [2], and life-threatening events as the most common traumatic event categories [3]. The word trauma is derived from the ancient Greek word for bodily damage wound (trauma) and began to develop a new metaphorical connotation in popular culture only in the late nineteenth century. Unlike the prevailing organic explanation and considering the abundance of childhood histories related to the severe abuse observed among patients suffering from unexplained somatic and emotional symptoms [4], French neurologists Jean-Martin Charcot and Pierre Janet hypothesized that the symptoms were caused by the idea of the trauma (i.e., the subjective perception of intensely distressing experiences), which provoked psychological and physical manifestations (hysteria) [5]. Currently, psychological trauma is typically defined as an unbearable and unavoidable threatening single or ongoing experience over which a person is helpless [6,7,8]. The traumatic extent of an event is determined not only by how harmful or threatening it is but also by how much it overwhelms a person’s ability to cope. The extent to which the fight/flight strategies are effective in the face of a threatening event determines trauma vulnerability [6]. Considering this evidence, childhood, representing a condition in which a person is completely dependent on their parents for safety and survival, may turn into an inherently vulnerable state if caregivers are, in addition to being openly abusive or threatening, inattentive and lacking their caring responsibility [9,10].

Regarding autobiographical memories, reduced recall of specific memories in trauma-exposed participants [11] has been linked to the presence of major depressive disorder, other depressive disorders (e.g., postnatal depression), bipolar disorder, obsessive compulsive disorder [12,13,14,15,16], post-traumatic stress disorder (PTSD), and acute stress disorder, as well as eating disorders, when compared to healthy controls [17]. Reduced specificity, according to longitudinal studies, also precedes diagnosis and predicts the progression of symptoms over time [18,19]. Reduced specificity can be caused by three pathways, according to the CaRFAX model [20]: Capture and Rumination, Functional Avoidance, and Impaired eXecutive control. As Griffith et al. [21] pointed out, each pathway can be linked to coping after a traumatic event. When a person is trying to recall memories of relatively benign events, for example, they may trigger a memory of a trauma, maybe due to semantic links (e.g., someone who has experienced sexual abuse may find that retrieval of other memories involving men evokes retrieval of memories regarding the abuse). Alternatively, if the trauma had a particularly detrimental impact on the individual’s perception of themselves and their surroundings, the person may recall more conceptual memories that support these negative ideas (e.g., attempts to retrieve memories of a social gathering may evoke retrieval of thoughts related to being unlovable). Capturing and ruminating on these thoughts can prevent the recall of the other, non-trauma-related memories, resulting in only a limited amount of information being retrieved. In addition, a person who has been exposed to trauma may avoid recalling specific parts of a trauma memory in order to lessen the emotional response that emerges with recalling these details, a process known as functional avoidance [10]. Given the semantic link between trauma-related and non-trauma-related memories, this tendency may also apply to other autobiographical memories [22,23]. As a result, a person may start to avoid the details of all memories due to the fear that they may be negatively valenced or that retrieving these details could elicit the recall of other trauma memories. Dysfunctional executive control, according to Williams et al. [17], can worsen these processes. For example, a person may show impaired memory specificity because their attention may be captured by other semantically similar and possibly more negative thoughts when recalling memories from their past. Furthermore, regarding the memories that they do retrieve, they may be less able, with respect to individuals with good executive control, to retain all of the details of a memory in mind at the same time and hence only report a limited level of detail. As a result, it is reasonable to predict that the specificity of memories retrieved for individuals who have experienced trauma will be diminished when compared to those who have not. The tendency to show this pattern of memory recollection, according to Williams et al. [17], is involved in the emergence and maintenance of psychopathology by affecting a person’s ability to solve problems and plan for the future based on past experiences, as well as how people regulate their emotions in the presence of significant life events.

The complexity of trauma narratives has been used as an index of fragmented memory in psychological trauma and PTSD [24]. Higher fragmentation soon after a traumatic event predicted more severe PTSD over time, according to prospective research (e.g., [25,26,27]). A paucity of pilot research works (*n* = 14 to *n* = 37) on PTSD [28,29,30,31] and on acute stress disorder (*n* = 15) [32] have examined trauma fragmentation pre- and post-treatment, and the results are mixed. Research that used the same narrative coding measure found that some measures of organization, such as planning and decision making [28] and disordered thinking [31], improved with therapy and were related to decreased anxiety. However, fragmentation (e.g., uncompleted thoughts) was either unrelated to symptom improvement [31] or did not change with treatment [28]. Other research works have found no association between fragmentation and changes in PTSD or acute stress disorder [29,32], and incoherence, a concept comparable to fragmentation, did not improve following therapy for childhood trauma [30]. A more recent study [2] investigated trauma and control narratives, comparing prolonged exposure (PE) and sertraline, respectively, pre- and post-treatment, in individuals with chronic PTSD. Despite the fact that sensory components increased with PE, there were no consistent variations in fragmentation between PE and sertraline or treatment responders and non-responders from pre- to post-treatment, suggesting that PE alone may be helpful for symptoms recovery but may not induce memory reprocessing and integration. Indeed, it has been proposed that a single underlying disintegrated event in memory networks could underpin the psychopathology of emotional distress [33] and that, even in the presence of symptoms recovery, when facing trauma-related cues again, they may act as new triggers that may re-precipitate symptoms. Strikingly, very recently, using multi-session functional magnetic resonance imaging (fMRI) [34,35], it has been shown that actual updating and re-integration of maladaptive memories occurred only through a positive emotion-focused strategy [36]. Indeed, the re-emergence of positive meaning and emotions at future retrieval occurred only if individuals focused on the positive aspects after an initial negative recall. Interestingly, the therapeutical process of eye movement desensitization and reprocessing (EMDR), an empirically validated treatment for psychological trauma, encompasses a specific focus related to an effort to find a desired positive cognition (PC) in order to promote an adaptive re-processing of an initial, trauma-related maladaptive negative cognition (NC) [37,38,39].

It has also been shown that human social experience, starting with primary social bonds, such as parent–child relationship, and extending to adulthood at both the dyadic and community or group level, plays a very important role in how an individual responds to trauma. There is mounting evidence that social support, social cognition, and attachment organization play a role in emotion regulation during severe stress and, more specifically, in preventing or recovering from PTSD (e.g., Refs [40,41,42,43]. In addition, youth that received trauma-focused cognitive-behavioral therapy (TF-CBT) built narratives with more structured thinking and an increased internal focus, both of which are regarded to be beneficial to traumatized children [44], as well as a higher resilience in the face of trauma represented by narratives with increased details of valued relational identities or newly developed relational identities [45]. More cohesive and ordered trauma narratives, on the other hand, were not linked to lower post-traumatic stress symptoms scores [44] measured with the clinical-administered PTSD scale for children and adolescents (CAPS-CA; [46]). These results suggest that the TF-CBT effects, though helpful at promoting narratives with more structured thinking, may not be associated with individuals’ symptoms-related disturbance.

It has been demonstrated that the subjective units of disturbance (SUD) scale may show a better association with individuals’ symptoms-related disturbance. Consistent with this, the patient’s level of depression, state anxiety, and distress from the impact of events were all significantly associated with initial SUD scores at the first session. The Clinical Global Impression (CGI; [47]) change score at termination was significantly associated with SUD final scores at the first session [48,49]. In addition, considering that actual updating and re-integration of maladaptive memories occurred only through a positive emotion-focused strategy [36], the PC of EMDR may be helpful in evaluating treatment efficacy. In fact, standard EMDR protocol assesses the validity of cognition (VoC) scale, which measures the believability of a suggested PC. The VoC scale uses a seven-point scale on which 1 indicates that the cognition feels completely false, and 7 indicates that it feels completely true, whereas the SUD scale assesses the intensity of negative affect on a ten-point scale on which 0 indicates no discomfort, and 10 indicates the highest level of discomfort or distress imaginable. It has been shown that SUD scale reduction promoted VoC scale increase and that both scale scores were associated with tension, depression, anger, fatigue, and confusion symptomatology improvement [50].

Group EMDR therapy is a well-proven form of treatment for traumatized children and adolescents [51,52,53,54]. To our knowledge, no study to date has evaluated the possible differential simultaneous effects of a group EMDR intervention on narrative complexity, specificity of autobiographical memories, event coding, SUD, and VoC in children aged 9–11 years. In addition, we aim to explore the existence of specific relationship patterns among the considered variables through a path analytic and supervised machine-learning (ML) approach. Here, our aim is to carry out a pilot study in non-clinical experimental and control samples regarding the exploratory investigation related to the appraisal of feasibility, duration, cost, and adverse events to be considered to plan future larger studies. The following were hypothesized: (a) narrative complexity, specificity of autobiographical memories, event coding, SUD, and VoC variables would improve in the experimental group after the group EMDR intervention but not in the control group; (b) SUD would negatively relate with VoC, narrative complexity, specificity of autobiographical memories, and event coding.

## 2. Materials and Methods

### 2.1. Trial Design

Our study is an interventional controlled trial with a randomized allocation, as indicated in the ClinicalTrials.gov Identifier NCT05319002 (https://clinicaltrials.gov/ct2/show/NCT05319002 (accessed on 11 April 2022); the “Hug the Child study”). The intervention strategy is based on a parallel assignment, with supportive care as the primary goal. The research was carried out in line with the Helsinki Declaration.

### 2.2. Participants

The following were the eligibility criteria: participants had to be between the ages of 9 and 11 years old, and both females and boys were accepted as healthy volunteers. Inclusion criteria were as follows: children aged 9 to 11 years old; children who have a reasonable understanding of spoken language and can follow simple commands; children and their parents who are willing to attend all intervention sessions; children and their parents who have a sufficient understanding of Italian. Exclusion criteria: concurrent inclusion in other intervention trials; child regularly practices EMDR intervention or other therapeutical interventions, such as CBT or meditation. The group EMDR program was implemented in a Pisa public elementary school. All of the study’s participants’ parents signed a written informed consent form.

### 2.3. Interventions

The “Hug the Child study,” which followed Template for Intervention Description and Replication (TIDieR) standards [55], was carried out from August 2021 to November 2021 using two arms: an experimental group that underwent a group EMDR intervention program and a control/no intervention group that followed their usual school activities. An experienced psychotherapist, who is also a certified EMDR supervisor, experienced in group EMDR, led the EMDR intervention program. The experimental group participated in a three-week consecutive group EMDR program that included weekly 90 min group sessions [56]. The group EMDR program was developed and implemented using methods documented in the literature [53,57].

### 2.4. Outcome Measures

The biopsychosocial interview [58] was used to obtain information regarding five sections: (1) family history; (2) life history; (3) school history; (4) romantic relationships; and (5) psychological issues and/or disorders history. Each section comprises about ten sub-sections with open-ended statements that serve as cues for the interviewer to document specific factual information (such as age, living arrangement, and relationship qualities) and also allows for additional information to be recorded. The child is encouraged to recall and reflect on events, while the interviewer pays attention to the child’s emotional responses, asking follow-up questions that may allow the child to make connections between events. Following the intake session and before the beginning of group EMDR sessions, the interview was used to elicit significant autobiographical memory narratives from the client’s life experiences. Throughout the experiment, autobiographical memory narratives were recorded with a microphone before the first group EMDR session and after the third session, and transcribed and analyzed a posteriori.

#### 2.4.1. Primary Outcome Measures

##### Narrative Complexity Level

Levels of narrative complexity were coded using the Coding System for Autobiographical Memory Narratives in Psychotherapy (CS-AMNP; [59]). During the coding process, the steps below were followed: (a) Topic definition. In order to isolate unique narrative components, the transcripts were divided into discrete topic sections, which are sections of the text with unique topic definition that avoid overlaps or repetitions in coding; (b) Narrative complexity coding. The causality, temporality, and outcome of narratives, as well as the representations of protagonists and antagonists and their emotional responses, were all used to determine the complexity of narratives. Each section’s narrative complexity was coded on a 5-point scale, with 5 representing the most complex and 1 indicating the least complex; (c) Defining autobiographical memory narratives. The last phase was related to finding narrative units that were about events that occurred at least one year ago and that the child had personally witnessed or experienced. The time range indicated aims to provide the memory narrative with enough time to connect to the long-term self’s enduring meaning and affective networks. Narrative complexity coding was carried out before the first group EMDR session and after the third session and was classified by three independent investigators (A.P., A.G. and M.M.). The Cohen Kappa coefficient was calculated to determine the inter-rater agreement. The coefficient was satisfactory (k = 0.87).

##### Specificity Level

Levels of specificity of autobiographical memories were coded using the Classification System and Scoring Manual for Self-Defining Autobiographical Memories (CS-SM-SDAM [60]). Autobiographical memories were coded as “specific” if they were a memory of a specific brief event with a unique occurrence, and with perceptual and sensory details, according to this classification method. More specifically, an autobiographical memory is a well-defined memory in which the participants describe their feelings about an event that has a distinct beginning and conclusion, and lasts no longer than 24 h. When a memory referred to events of longer duration or recurring experiences, they were labeled as general. Specificity level coding was carried out before the first group EMDR session and after the third session and was classified by three independent investigators. The Cohen Kappa coefficient was satisfactory (k = 0.84).

##### Event Coding

Recalled events were coded using the Manual of Coding Events in Self-Defining Memories (MCE-SDM; [61]). MCE-SDM classified autobiographical memories into seven categories: (1) life-threatening events; (2) recreation/exploration; (3) relationship; (4) achievement; (5) guilt/shame; (6) drug, alcohol, or tobacco usage; or (7) unclassifiable. Each narrative could only be assigned to one of the categories. Event coding was carried out before the first group EMDR session and after the third session and was classified by three independent investigators. The Cohen Kappa coefficients were satisfactory for all the seven categories (k ranged from 0.82 to 0.89).

##### Subjective Unit of Distress (SUD)

SUD is a 0-to-10-point self-reporting scale, and participants are asked to rate their level of discomfort or anxiety. The lack of distress is indicated by a score of 0, and the maximum amount of distress is indicated by a score of 10 [38]. This measure has been frequently used in research, with studies confirming its validity and reliability [62]. SUD scale was assessed before the first group EMDR session and after the third session. In this study, Cronbach’s α for the SUD scale was 0.89.

##### Validity of Cognition (VoC)

The VoC indicates to what extent the person believes that the chosen positive cognition is trustworthy. It is a self-reporting scale and provides an individual’s judgment of her or his own cognition, similar to the SUD scale. The participant’s self-recognition is evaluated, quantified, and indicated using a 7-point rating scale ranging from 1 to 7. A score of 1 indicates that the participant does not believe at all in that particular positive cognition, whereas a score of 7 indicates that the participant fully believes in that specific positive cognition [38]. This measure has been frequently used in research as well, with studies confirming its validity and reliability [62]. VoC scale was assessed before the first group EMDR session and after the third session. In this study, Cronbach’s α for the VoC scale was 0.87.

#### 2.4.2. Secondary Outcome Measures

##### Autobiographical Memory Definition

Definition of autobiographical memories was coded using the third phase of CS-AMNP coding [59], in particular during the aforementioned “defining autobiographical memory narratives” phase. Briefly, it is related to finding narrative units that were about events that occurred at least one year ago and that the child had personally witnessed or experienced. Autobiographical memory definition was carried out before the first group EMDR session and after the third session and was classified by three independent investigators. The Cohen Kappa coefficient was satisfactory (k = 0.86).

##### Integration Level

The level of memory integration was coded using CS-SM-SDAM [60]. Memories were classified as “integrated” if they were related to a value or meaning that the individual derived from his or her experience, according to Singer and Blagov’s CS-SM-SDAM. Meaning directly linked to the self and meaning linked to others or the environment were identified as two categories of integrative meaning. If the memory was purely narrative and not clearly related to learning about oneself, others, or the environment, it was classified as “non-integrated”. The integration level was carried out before the first group EMDR session and after the third session and was classified by three independent investigators. The Cohen Kappa coefficient was satisfactory (k = 0.87).

### 2.5. Sample Size

In order to conduct our pilot study [63,64], a minimum sample size of 12 per group was considered [65]. Among students of public primary schools in Pisa, 89 were evaluated for eligibility. In total, 57 students who were screened for eligibility declined to be enrolled in the intervention, and 5 did not participate because the children and their parents were unable to attend all of the sessions. The study enrolled a total of 27 subjects for randomized assignment.

### 2.6. Randomization

Using a computer-generated basic randomization sequence, subjects were randomly assigned in a 1:1 ratio to either the experimental intervention group (group EMDR) or the no-intervention group (daily normal school activities). A statistician who was not otherwise involved in the study and had no interaction with the study participants performed randomization following the baseline assessment. The allocation was and will be blinded to the outcome evaluators, and participants were informed not to reveal their group assignment to them. The psychologists that carried out the intervention were not the same as the ones who assessed the results.

### 2.7. Statistical Analyses

All basic statistical analyses were performed with SPSS^®®^ 27 (IBM Corp., Armonk, NY, USA), SigmaPlot^®®^ 14 (Systat software, Chicago, IL, USA), AMOS^®®^ 27 (Analysis of MOmentum Structures; IBM Corp., Armonk, NY, USA), and Weka 3.8.6 data mining software [66,67]. The Shapiro–Wilk test was performed to verify the non-normality of the distributions. In order to compare age between control and group EMDR samples, we applied a Mann–Whitney rank sum test (MWRST), while to compare gender frequency, we applied Barnard’s exact test (more powerful than Fisher’s exact test [68]). In order to compare categorical outcome variables, before and after treatment, we used McNemar’s test with Yates correction [69]. For comparisons between groups, before treatment, Friedman’s two-way analysis of variance on ranks (F-tw-ANOVA) with Dunnett post hoc group rank sums comparisons against a control group were used; meanwhile, for comparisons within groups, after treatment, a F-tw-ANOVA with Tukey post hoc rank sums comparisons were used. In order to identify the best models for predicting the narrative complexity level, SUD and VoC scales, the general linear model (GLM) regression analysis was used, and in order to control for multicollinearity, the variance inflation factor (VIF) and the condition number (K(A) = ‖A‖‖A^−1)^‖), a measure of the sensitivity of the parameter estimates to small changes in the data matrix [70,71], were calculated. For the narrative complexity level, SUD and VoC scales to be predicted as a criterion, the model showing adjusted R^2^ was considered.

In order to explore and confirm a possible path model, AMOS^®®^ 27.0 was employed. The *p* values reported were two tailed, and a *p* value < 0.05 was considered significant. Before performing path analysis, we analyzed the relationships between the variables. The absolute fit indices utilized in this study were χ^2^ and the root mean square error of approximation (RMSEA); the incremental fit indices used in this investigation were the comparative fit index (CFI) and the Tucker–Lewis index (TLI). CFI and TLI values of 0.90 or higher, and RMSEA values of 0.06 or lower, were considered a “good fit.” A better fit is indicated by χ^2^ values that are closer to zero. Since χ^2^ is sensitive to the sample size employed in the model fit analysis, it was not suggested as a model fit judgement [72]. Hence, it was just reported in this study but not used as a fit statistic. We employed the following model fit criteria [73]: TLI and CFI: ≥0.90 indicated acceptable fit, ≥0.95 indicated excellent fit; RMSEA: ≤0.08 indicated acceptable fit, ≤0.06 indicated excellent fit, and its 90% confidence interval (CI) was reported.

The following predictors were utilized in building the ML models to evaluate the effectiveness of event coding and narrative specificity level: narrative complexity and specificity levels, SUD and VoC scales to predict event coding; narrative complexity level, event coding, SUD and VoC scales to predict narrative specificity level. The technique of k-fold cross validation was applied. The k = 10 method was used, in which the value for k is fixed to 10, a value that was found to have a low bias through experimentation.

## 3. Results

### 3.1. Group Comparisons

**Figure 1:**
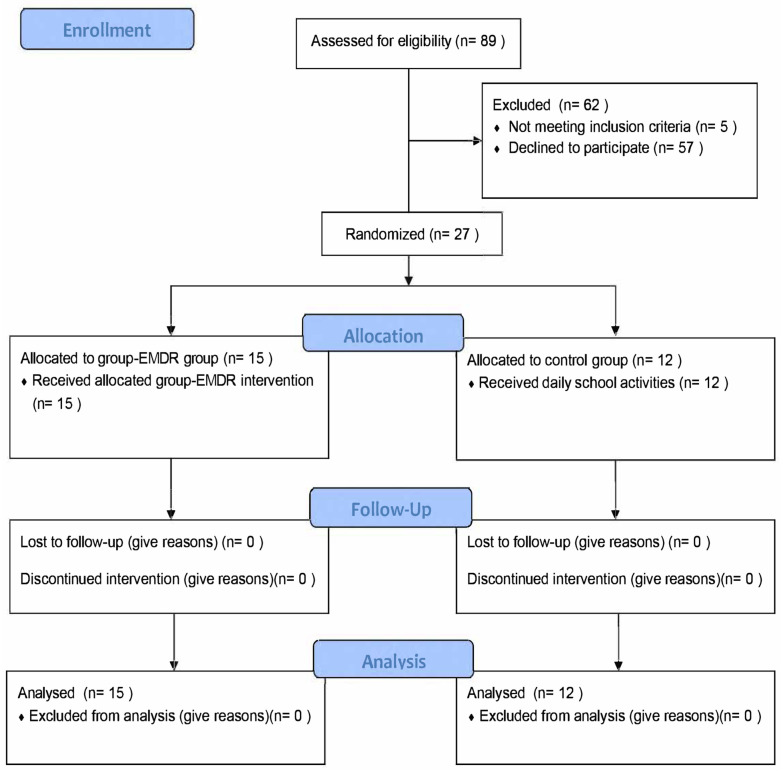
CONSORT flow diagram of the study.

As reported in the CONSORT flow diagram [74] of the study in Figure 1, 27 subjects (15 females, 55.55%; mean age = 10.85, SD = 0.38) belonging to two fifth-year primary school classes were included in the study. The control group was composed of 12 subjects (7 females, 58.33%), and the EMDR group was composed of 15 subjects (8 females, 53.33%).

As a first step, we compared gender frequency and age between control and group EMDR samples to evaluate gender and age homogeneity among groups. Barnard’s exact test revealed that gender frequency was not significantly different (*p* = 0.826) between the two groups, while MWRST showed that age was not significantly different (*p* = 0.177) as well. Thus, the control and EMDR groups were homogeneous regarding gender and age. Hence, we compared the scores generated by participants between the two groups (before group EMDR intervention) and within the two groups (after group EMDR intervention) regarding scales of the study measures. Regarding pre-intervention baseline measures, McNemar’s test with Yates correction revealed that event coding and specificity level, as well as autobiographical memory definition and integration level (Supplementary Table S1), were not significantly different between the control and EMDR groups (event coding: *p* = 1; specificity level: *p* = 0.45; autobiographical memory definition: *p* = 1; integration level: *p* = 1), while Dunnett’s post hoc analysis of F-tw-ANOVA revealed that control and EMDR samples did not show scores that were significantly different for narrative complexity level (*p* = 0.999), SUD (*p* = 0.889), and VoC (*p* = 0.225) scales. Conversely, comparing post-intervention measures, McNemar’s test with Yates correction revealed that event coding and specificity level were significantly different between the control and EMDR groups (event coding: *p* = 0.005; specificity level: *p* = 0.008), while Dunnett’s post hoc analysis of F-tw-ANOVA revealed that control and EMDR samples showed a significant difference regarding narrative complexity level (*p* = 0.003), SUD (*p* = 0.008), and VoC (*p* = 0.01) scales (Table 1). Autobiographical memory definition and integration level were not significantly different between the control and EMDR groups (autobiographical memory definition: *p* = 1; integration level: *p* = 1) (Table 1).

In order to identify the effects of the group EMDR intervention on the experimental group, we compared separately the control and EMDR groups, before and after group EMDR intervention. As expected, McNemar’s test with Yates correction showed that the control sample, after the daily routine school activities, did not show scores that were significantly different for event coding (*p* = 0.617) and specificity level (*p* = 0.54), as well as autobiographical memory definition (*p* = 0.617) and integration level (*p* = 0.617) (Supplementary Table S2). In addition, Tukey’s post hoc test of F-tw-ANOVA revealed that the control sample, after the daily routine school activities, showed scores that were not significantly different for narrative complexity level (*p* = 0.395), SUD (*p* = 0.129), and VoC (*p* = 0.34) scales. Conversely, McNemar’s test with Yates correction showed that the EMDR sample, after the group EMDR intervention, showed scores that were significantly different for event coding (*p* = 0.019) and specificity level (*p* = 0.008). In addition, Tukey’s post hoc test of F-tw-ANOVA revealed that the EMDR sample, after the group EMDR intervention, showed scores that were significantly different for narrative complexity level (*p* = 0.005), SUD (*p* = 0.001) and VoC (*p* = 0.005) scales (Table 2). Autobiographical memory definition and integration level were not significantly different within the EMDR group, after the group EMDR intervention (autobiographical memory definition: *p* = 0.773; integration level: *p* = 0.289) (Table 2).

### 3.2. GLM Regressions

None of the observed variables have to be normal to implement linear regression analysis; however, the errors after modeling should be normally distributed to draw a valid conclusion by hypothesis testing [75,76]. Hence, in order to identify the best models predicting each of the interval variables, we carried out the GLM regression analysis, and all the interval variables that showed a significant effect after EMDR intervention were considered. The VIF was computed for each predictor and fell within the range (1.01–1.26), which is considered as evidence of a lack of substantial multicollinearity [77]. Condition number was 8.149, and values above 30 are considered to be indicative of multicollinearity [70,71]. Results of the regression analyses predicting each of the interval variables for the post-intervention EMDR group are reported in Supplementary Table S3. First, we evaluated which of the interval variables were able to predict narrative complexity levels. VoC (β = −0.261, *p* = 0.554) as well as SUD (β = −0.0242, *p* = 0.853) scales were not significant predictors of narrative complexity levels. When inspecting which of the interval variables were able to predict the VoC scale, narrative complexity levels and SUD scale were not able to be significant predictors (β = −0.115, *p* = 0.554 for narrative complexity levels; β = −0.134, *p* = 0.1 for SUD scale). SUD scale was predicted neither by narrative complexity level (β = −0.124, *p* = 0.853) nor by VoC (β = −1.562, *p* = 0.1).

### 3.3. Path Analysis

In order to compare different models to determine which one best fits the data and to analyze models that are more complex and realistic than multiple regression [78], path analytic models were tested using AMOS^®®^ 27 for EMDR group post-intervention to evaluate if there was a possible particular association among the interval and categorical variables [79]. Path analysis is a particular type of structural equation modeling (SEM) that is itself a GLM development. GLM is a second generation of the method of data analysis, which depends on a momentum structural relationship existing among variables of interest. SEM can be carried out using software such as AMOS^®®^ 27 [80]. Accordingly, we used the Bayesian estimator in AMOS^®®^ 27 for data sets containing variables with fewer than five categories [79] and that fail to satisfy normality assumptions [81,82], as well as RMSEA, CFI, and TLI [83].

**Figure 2:**
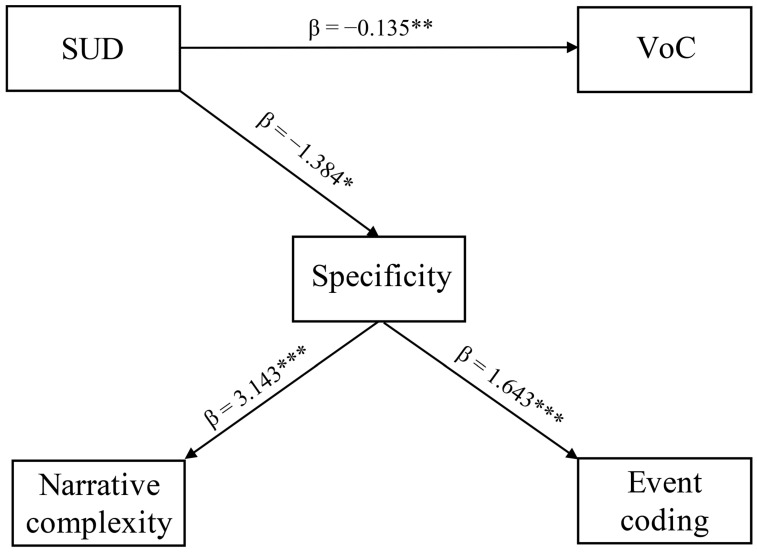
Path analytic model showing SUD scale as a predictor of both VoC scale and specificity level, and the latter, in turn, acting as a predictor of both narrative complexity levels and “relationship” event coding. * *p* < 0.05, ** *p* < 0.01, *** *p* < 0.001.

In the EMDR group post-intervention, a model showing SUD scale as a predictor of both the VoC scale and specificity level, and the latter, in turn, acting as a predictor of both narrative complexity levels and “relationship” event coding, was found to achieve the highest fit among all of the models evaluated through the combinations of the considered variables (χ^2^ (6) = 2.301, *p* = 0.89). However, we further inspected specific RMSEA, CFI, and TLI as fit indices. We found that the SUD scale was able to significantly predict both the VoC scale (β = −0.135, *p* = 0.007, SE = 0.071) and specificity level (β = −1.384, *p* = 0.011, SE = 0.021). Furthermore, specificity level was found to be a significant predictor of both narrative complexity level (β = 3.143, *p* < 0.001, SE = 0.947) and “relationship” event coding (β = 1.643, *p* = 0.001, SE = 0.632). Overall, the SUD scale was found to be the unique predictor of both the VoC scale and specificity level, and, in turn, specificity level was found to be the unique predictor of both narrative complexity level and “relationship” event coding (Figure 2) (CFI = 0.997, TLI = 0.981, RMSEA = 0.023 [0.022; 0.026]).

### 3.4. Machine-Learning Analysis

In order to compare path analysis and ML algorithms [84] and to obtain a hierarchical classification of our variables, elucidating the possible mechanism of action of group EMDR intervention on the examined variables, an ML analysis was carried out. Five different classifiers were compared: naïve Bayes (*n*° correct classification: 14/15; accuracy: 93.33%; area under ROC curve: 0.855), simple logistics (*n*° correct classification: 13/15; accuracy: 86.67%; area under ROC curve: 0.781), logistic regression (*n*° correct classification: 13/15; accuracy: 86.67%; area under ROC curve: 0.714), Hoeffding tree (*n*° correct classification: 13/15; accuracy: 86.67%; area under ROC curve: 0.874), random tree (*n*° correct classification: 14/15; accuracy: 93.33%; area under ROC curve: 0.891). Results related to ML classifiers are shown in Table 3.

## 4. Discussion

Our study demonstrates that group EMDR intervention may be able to improve SUD and VoC scales, narrative complexity, and specificity levels, as well as “relationship” event coding in the experimental group, with respect to control group following daily routine school activities. Regarding SUD levels reduction predicting VoC levels increase, it has already been shown that alternating tactile bilateral stimulation (BLS) with a vibration machine, with respect to a non-BLS condition, promoted the accessibility to PC [85]. Multi-channel near-infrared spectroscopy showed that, in response to BLS, a significant increase in oxyhemoglobin was detected in the right superior temporal sulcus (STS), and a decrease in the wide bilateral areas of the prefrontal cortex (PFC) was observed. The significant activation of the right STS in response to BLS, which is linked to memory representation, suggests that BLS could aid in the recollection of more representational memories related to pleasurable feelings. In addition, the significant activity decrease in the PFC, which is associated with emotion regulation, suggested that BLS may promote relaxation and pleasant/soothing feelings [85]. Since it has been shown that in order to carry out an actual updating and re-integration of maladaptive memories and to promote a re-emergence of positive meaning and emotions at future retrieval individuals had to focus on the positive aspects after an initial negative recall [36], SUD scale reduction and VoC scale increase may be a prerequisite to access narrative specificity, as shown by the path analytic model and hierarchical ML classification.

Regarding the effects of SUD scale on narrative specificity, past research investigated SUDs and linguistic change in adults with PTSD to determine whether SUDs and linguistic change were associated with PTSD symptom improvement. Trauma narratives in patients with PTSD have been found to be cognitively and temporally disorganized but with increased emotional references [86]. During four 30 min writing sessions, 29 African American participants with PTSD underwent the assessment of PTSD symptoms, SUD scale, narratives (through the linguistic inquiry word count, LIWC [87], for changes in rates of affective, cognitive, and temporal word usage), heart rate, and skin conductance. Writing sessions 1 and 2 were 12 h apart, while sessions 3 and 4 were 12 h apart a week later. Results showed that participants exhibited significant PTSD symptom improvement, decreased SUD scale after four sessions writing about a traumatic event, as well as decreased negative words and more time-related words in their trauma narratives after completing the series. Hence, trauma narrative evolved in a well-defined memory in which the participants described their feelings about the event, having a distinct beginning and conclusion, and lasting no longer than 24 h [86]. Thus, the decrease in SUD scale was directly associated with an increase in narrative specificity.

The definition of a specific trauma narrative as a well-defined memory with participants describing their feelings about the event, having a distinct beginning and conclusion, and lasting no longer than 24 h recapitulates the definition of an episodic memory [88], and narrativity has been proposed as an indicator of episodic memory strength when people discuss their past [89]. In addition, referential activity, the extent to which words convey a speaker’s experience of being present in the event being described, and narrative temporal sequence have been independently hypothesized to indicate episodic memory strength [90,91]. Interestingly, temporality, causality, as well as the representations of protagonists and antagonists and their emotional responses, have been identified as criteria for narrative complexity coding in CS-AMNP [59]. Hence, it could be hypothesized that the processing of a traumatic memory, through the reduction in SUD scale and the increase in VoC scale, may gradually promote the evolution of the traumatic memory into an actual specific episodic memory, through fostering the development of narrative complexity, which is then stored as a past autobiographical memory.

Interestingly, it has been shown that narrative memories are constructed by the hippocampus across distant events [92]. Patterns of hippocampal activity, including activity at event boundaries, were more similar between distant events that formed one cohesive narrative during encoding of fictional stories compared with overlapping events from unrelated narratives. One day later, the hippocampus preferentially supported detailed recall of coherent narrative events, through reinstatement of hippocampal activity patterns from encoding [92]. Remarkably, during EMDR protocol, patients may start processing an event related to a NC and then recall events that are temporally very distant and that may be related to NC but also to PC. In addition, during processing, narratives may show an increase in cooperative relational details. Overall, PC and relational details may contribute to overcoming NC and creating a coherent narrative [38]. In fact, after group EMDR intervention, we found that narratives that could be coded as “relationship” showed a significant increase, and, in accordance with this, it has been suggested that a specific focus on cooperation and trusting behavior could provide a treatment for interpersonal trauma [93].

An asymmetry in how adults use positive and negative information to make sense of their world has been proposed; specifically, adults may show a negativity bias or a propensity to pay attention to, learn from, and use negative information far more than positive information across a variety of psychological situations and tasks. This bias is thought to serve important evolutionary functions, but its developmental occurrence and ontogenetic emergence have never been investigated [94]. Interestingly, along the information processing leading to narrative specificity and complexity, EMDR may operate through the homeostatic regulation of negativity. Negativity bias may serve evolutionarily protective functions, but early developmental traumatic experiences may severely increase the negativity bias in adulthood [95]. In fact, negativity bias is thought to emerge early in development, and it has been observed in studies on infant social referencing as well as other developmental areas; furthermore, negativity bias is robust and active in the emotional domain in humans aged 12 months and older [94]. Accordingly, we proposed the existence of a sensitive period related to the development of safety during the first year of life [96,97], and early traumatic experiences may severely impinge on this period, generating traumatic autobiographical memories with elevated SUD and fragmented narratives [2].

## 5. Strengths and Limitations

Our study shows some strengths: (a) investigation of autobiographical narratives with validated tools; (b) evaluating the effects of a group EMDR intervention on autobiographical narratives using a randomized controlled design; (c) we analyzed specific relationships among SUD and VoC scales, specificity and narrative complexity levels and “relationship” event coding using both path analytic and supervised machine-learning approach.

Our findings, however, should be considered in light of the following limitations: (a) as a pilot study, our samples were relatively small. Further studies should replicate our findings in larger samples; (b) participants were self-selected. This might limit the generalizability of our conclusions; (c) in order to achieve narrative analysis, we included tools such as CS-AMNP [59], CS-SM-SDAM [60], and the MCE-SDM [61] in our study. Future research could replicate our results using other tools for narrative analysis, such as the LIWC [87]; (d) self-reported data tend to inflate associations among variables; (e) all narratives are co-constructed [98], and we did not investigate how the therapeutical alliance or the relationship with the therapist may have influenced the narrative construction. A strong alliance combined with trauma-focused work was found to predict the outcome in previous research [99], and it is possible that a solid therapeutical relationship may facilitate narrative construction. Future research should investigate the role of the therapeutical alliance in narrative construction; (f) though other previous randomized controlled trials have focused on non-clinical samples (e.g., Ref. [100]), our study recruited a non-clinical sample of primary school children, so any extrapolation of the findings to clinical populations should be made with caution. However, considering that our intervention with group EMDR was significant with a relatively small non-clinical sample, whose levels of distress were generally lower than those shown by clinical samples, we expect that a group EMDR intervention would be highly beneficial for clinical samples.

## 6. Conclusions

Despite these limitations, our study found that a group EMDR program improved SUD and VoC scales, narrative complexity level, narrative specificity level and promoted “relationship” event coding in a sample of primary school children (9–11 years old) as compared to a non-active control group. Our findings support the use of group EMDR in primary school children in order to prevent the fragmentation of autobiographical memories’ narrative [101]. More importantly, group EMDR may represent an effective strategy as an early intervention and prevention of the development of psychological disorders in the long term following situations of acute stress [102]. Interestingly, miRNA-29a was identified as a response predictor for EMDR integrative effects [103] (while a mindfulness intervention [104] was found to improve cognitive function [105] by increasing the neuronal expression of miRNA-29c [106]), so a group EMDR intervention may represent an effective strategy to promote integration in clinical [52] and non-clinical individuals [107].

## Figures and Tables

**Table 1:** Group comparisons among the study measures between EMDR (*n* = 15) and control (12) samples assessed pre-intervention and post-intervention.

Variable	Control—Pre	EMDR—Pre	*p*	Control—Post	EMDR—Post	*p*
1. Event coding (Relationship; *n*°/%)	2 (16.67%)	3 (20%)	=1	1 (8.33%)	10 (66.67%)	=0.005
2. Specificity level (Specific; *n*°/%)	7 (58.33%)	6 (40%)	=0.45	5 (41.67%)	14 (93.33%)	=0.008
3. Narrative complexity level	2.75 (1.14)	2.6 (0.91)	=0.999	2.42 (1.08)	3.93 (1.22)	=0.003
	2.5 [1.25]	3 [0.5]		2.5 [1.25]	4 [1.5]	
4. Subjective unit of distress (SUD)	9.5 (1)	9.53 (0.74)	=0.884	8.42 (2.27)	5.47 (3.07)	=0.008
	10 [0.25]	10 [1]		9.5 [2.25]	5 [5.5]	
5. Validity of cognition (VoC)	5.5 (1.45)	4.8 (1.57)	=0.225	5 (1.65)	6.4 (0.91)	=0.01
	6 [1.25]	4 [2.5]		5 [2]	7 [1]	

Note: *p* = *p*-value resulting from McNemar’s Test with Yates correction for rows 1–2 and from Dunnett’s post hoc test from Friedman’s two-way analysis of variance on ranks for rows 3–5; 1. Event coding from the Manual for Coding Events in Self-Defining Memories; 2. Specificity level from the CS-SM-SDAM; 3. Narrative complexity level from the CS-AMNP; 4. SUD scale from EMDR protocol; 5. VoC scale from EMDR protocol. Frequency and percentage (in brackets) are shown for rows 1–2. Mean and standard deviation (in brackets) and median and interquartile range (in square brackets) are shown for rows 3–5.

**Table 2:** Group comparisons among the study measures within EMDR (*n* = 15) and control (12) samples assessed pre-intervention and post-intervention.

Variable	Control—Pre	Control—Post	*p*	EMDR—Pre	EMDR—Post	*p*
1. Event coding (Relationship; *n*°/%)	2 (16.67%)	1 (8.33%)	=0.617	3 (20%)	10 (66.67%)	=0.019
2. Specificity level (Specific; *n*°/%)	7 (58.33%)	5 (41.67%)	=0.54	6 (40%)	14 (93.33%)	=0.008
3. Narrative complexity level	2.75 (1.14)	2.42 (1.08)	=0.395	2.6 (0.91)	3.93 (1.22)	=0.005
	2.5 [1.25]	2.5 [1.25]		3 [0.5]	4 [1.5]	
4. Subjective unit of distress (SUD)	9.5 (1)	8.42 (2.27)	=0.129	9.53 (0.74)	5.47 (3.07)	=0.001
	10 [0.25]	9.5 [2.25]		10 [1]	5 [5.5]	
5. Validity of cognition (VoC)	5.5 (1.45)	5 (1.65)	=0.34	4.8 (1.57)	6.4 (0.91)	=0.005
	6 [1.25]	5 [2]		4 [2.5]	7 [1]	

Note: *p* = *p*-value resulting from McNemar’s Test with Yates correction for rows 1–2, and from Tukey’s post hoc test from Friedman’s two-way analysis of variance on ranks for rows 3–5. 1. Event coding from the Manual of Coding Events in Self-Defining Memories; 2. Specificity level from the CS-SM-SDAM; 3. Narrative complexity level from the CS-AMNP; 4. SUD scale from EMDR protocol; 5. VoC scale from EMDR protocol. Frequency and percentage (in brackets) are shown for rows 1–2. Mean and standard deviation (in brackets) and median and interquartile range (in square brackets) are shown for rows 3–5.

**Table 3:** Accuracy, area under ROC curve, and number of correct classifications resulting from different machine-learning classifiers, using 10-fold cross validation, for group EMDR (*n* = 15) sample assessed post-intervention.

Machine-Learning Classifier	Accuracy (%)	Area under ROC Curve	Correct Classification (*n*° Correct/Total)
1. Naïve Bayes	93.33	0.855	14/15
2. Simple logistics	86.67	0.781	13/15
3. Logistic regression	86.67	0.714	13/15
4. Hoeffding tree	86.67	0.874	13/15
5. Random tree	93.33	0.891	14/15

Note: ROC curve = receiver operating characteristic curve.

## Data Availability

The data presented in this study are available on request from the corresponding author. The data are not publicly available due to privacy issue.

## Supplementary Materials

The following supporting information can be downloaded at: https://www.mdpi.com/article/10.3390/ijerph19137684/s1, Table S1: Group comparisons among the study measures between EMDR (*n* = 15) and Control (12) samples assessed pre-intervention and post-intervention. Table S2: Group comparisons among the study measures within EMDR (*n* = 15) and Control (12) samples assessed pre-intervention and post-intervention. Table S3: GLM regression analyses predicting narrative complexity level, SUD and VoC score from the other interval scales for group EMDR sample post-intervention (*n* = 15).
