# Supplementary material for: Randomized Trial on the Effects of a Group EMDR Intervention on Narrative Complexity and Specificity of Autobiographical Memories: A Path Analytic and Supervised Machine-Learning Study
Source: Int J Environ Res Public Health. 2022 Jun 23;19(13):7684. doi: 10.3390/ijerph19137684 (PMC9265795; doi:10.3390/ijerph19137684)
Supplement: Supplementary file 1 [file ijerph-19-07684-s001.zip › ijerph-1763746-supplementary.pdf]

Supplementary Table S1

Group comparisons among the study measures between EMDR (n = 15) and Control (12) samples assessed pre-intervention and post-intervention.

Variable	Control - Pre	EMDR - Pre	*p*	Control - Post	EMDR - Post	*p*
1. Autobiographical memory definition (Autobiographical; n°/%)	12 (100 %)	14 (93.33 %)	= 1	11 (91.67 %)	13 (86.67 %)	= 1
2. Integration level (Non-Integrated; n°/%)	12 (100 %)	15 (100 %)	= 1	11 (91.67 %)	13 (86.67 %)	= 1

Note: *p* = *p*-value resulting from McNemar's Test for rows 1-2. 1. Autobiographical memory definition from the Coding System for Autobiographical Memory Narratives in Psychotherapy (CS-AMNP); 2. Integration level from the Classification System and Scoring Manual for Self-Defining Autobiographical Memories (CS-SM-SDAM). Frequency and percentage (in brackets) is shown for rows 1-2.

Supplementary Table S2

Group comparisons among the study measures within EMDR (n = 15) and Control (12) samples assessed pre-intervention and post-intervention.

Variable	Control - Pre	Control - Post	*p*	EMDR - Pre	EMDR - Post	*p*
1. Autobiographical memory definition (Autobiographical; n°/%)	12 (100 %)	11 (91.67 %)	= 0.617	14 (93.33 %)	13 (86.67 %)	= 0.773
2. Integration level (Non-Integrated; n°/%)	12 (100 %)	11 (91.67 %)	= 0.617	15 (100 %)	13 (86.67 %)	= 0.289

Note: *p* = *p*-value resulting from McNemar's Test with Yates correction for rows 1-2. 1. Autobiographical memory definition from the Coding System for Autobiographical Memory Narratives in Psychotherapy (CS-AMNP); 2. Integration level from the Classification System and Scoring Manual for Self-Defining Autobiographical Memories (CS-SM-SDAM). Frequency and percentage (in brackets) is shown for rows 1-2.

Supplementary Table S3

GLM regression analyses predicting narrative complexity level, SUD and VoC score from the other interval scales for group EMDR sample post-intervention (n = 15).

Predictor	β	*t*	*p*
Criterion: Narrative complexity level			
Adjusted R^2 = .175			
VoC	-.261(.429)	-.609	.554
SUD	-.0242(.127)	-.190	.853
Criterion: VoC			
Adjusted R^2 = .231			
Narrative complexity	-.115(.189)	-.609	.554
SUD	-.134(.0751)	-1.781	.1
Criterion: SUD			
Adjusted R^2 = .21			
Narrative complexity	-.124(.653)	-.19	.853
VoC	-1.562(.877)	-1.781	.1

Note: Standard errors in parentheses; SUD = Subjective Unit of Distress; VoC = Validity of Cognition.
